# A Modified Bilaminar Technique with the Use of a Fibrin-Fibronectin System for a Single Gingival Recession: A Case Report with a Follow-Up of 3 Years

**DOI:** 10.1155/2020/3892753

**Published:** 2020-09-30

**Authors:** Michele Perelli, Paolo Giacomo Arduino, Mario Semenza, Roberto Abundo, Hector Sarmiento

**Affiliations:** ^1^Indipendent Researcher, Turin, Italy; ^2^Department of Surgical Sciences, CIR-Dental School, University of Turin, Turin, Italy; ^3^Indipendent Researcher, Sant'Angelo Lodigiano, Italy; ^4^Indipendent Researcher, New York City, USA

## Abstract

This case report described a modified bilaminar technique for treating a single gingival recession. Patient presented a gingival recession in a maxillary canine. Tooth was in a buccally prominent position and soft keratinized tissue apical to the recession was reduced but still present. A split-full-split thickness trapezoidal flap was designed. Root's surface was prepared with curettes. Epithelial-connective tissue graft was harvested from the palate with reduced dimension. After deepithelialization, the graft was placed with a fibrin-fibronectin system at the maximum root coverage level, and the flap coronally advanced and sutured. At 3-year follow-up control, the free gingival margin was still stable at the postsurgery position, with a thicker biotype corresponding to the grafted area, with no probing and a suitable aesthetic result.

## 1. Introduction

Gingival recession consists in the apical shift of the free gingival margin with the consequent exposure of the cementum enamel junction and the root surface [1]. This gingival loss may determine aesthetic problems, as well as dental hypersensitivity, noncarious cervical lesions, or radicular caries.

Different authors have reported a relationship between the inflammatory state of the marginal soft tissue and the amount of keratinized soft tissue, demonstrating the need of a minimum quantity of such tissue to permit a proper plaque control [2, 3]. In addition, patients' aesthetic concerns, and their perception, have currently increased, and different surgical techniques have been developed to reach root coverage (complete when is possible) and to increase keratinized marginal soft tissue [4–7]. Among them, both coronally advanced flap (CAF) [8–10] and connective tissue graft (CTG) [11–15] have shown suitable and predictable results. Different studies have demonstrated how the addition of a CTG to a coronally advanced flap (called “bilaminar technique”) may act as a flap stabilizer thickening of the marginal soft tissue, resulting in a more predictable and stable result of root coverage especially in the long-term follow-up [16, 17]. Since the first reported bilaminar technique [12], various surgical approaches have been proposed to reduce graft dimension, patient palatal discomfort, and morbidity in the second surgical area.

Aim of this case report was to describe a modified bilaminar technique, with a reduced mesiodistal dimension of the CTG and bonded to the radicular dentin by means of a fibrin-fibronectin system. Over a follow-up period of 3 years, this technique has demonstrated to be effective in reaching a good root coverage, a marginal soft tissue stability and an increased connective volume.

## 2. Case Presentation

**Figure 1:**
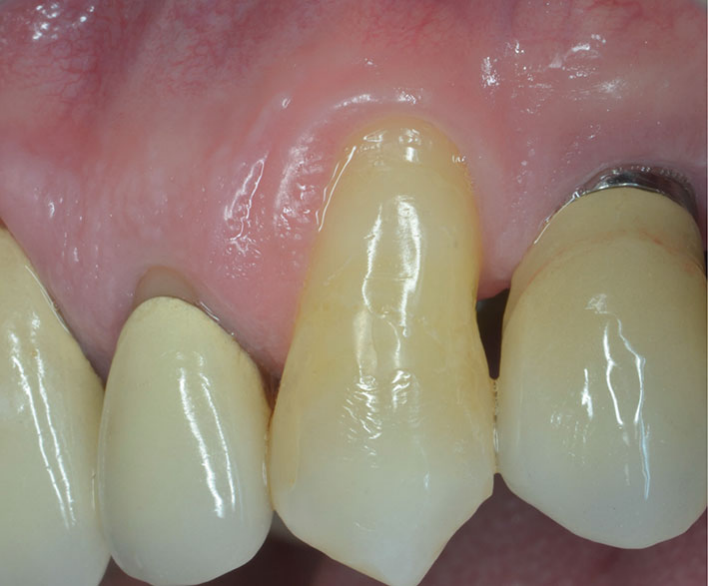
Left maxillary canine presenting a RT2 gingival defect.

**Figure 2:**
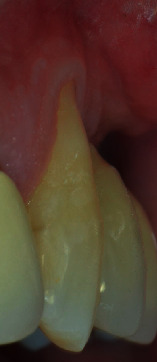
Lateral view: noncarious cervical lesion with composite restoration is evident (note the radicular concavity).

A 53-year-old male, not smoker, presenting a recession type 2 (RT2) gingival defect [18] in correspondence of the maxillary left canine, was referred for a periodontal evaluation (Figures 1 and 2). His main complaint was tooth hypersensitivity and scar of losing all the gingival support. He did not present any medical contraindication for periodontal surgery. The treatment plane was aimed at partial root coverage and marginal soft tissue augmentation. After signing a tailored written consent, he firstly received a nonsurgical periodontal therapy, including oral hygiene instructions and supra- and subgingival scaling, by an experienced dental hygienist. Patient was also instructed about oral hygiene maintenance at home; instructions included modified Bass technique with soft brushes, in order not to damage soft marginal tissue. At 1-month control, the full-mouth bleeding score (FMBS) and the full-mouth plaque score (FMPS) indexes were both ≤25%.

**Figure 3:**
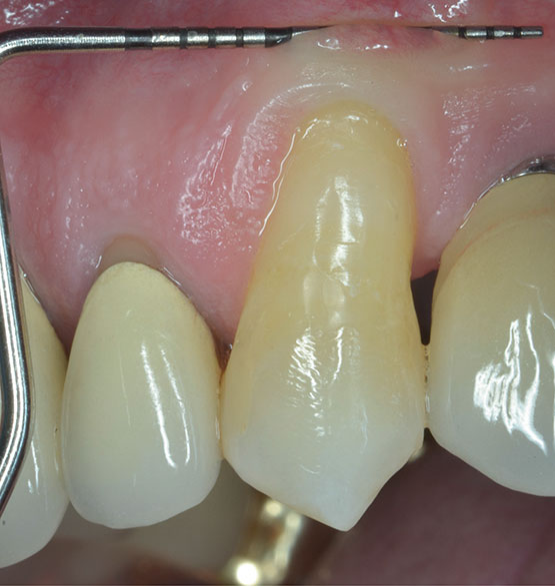
A minimum amount of keratinized tissue apical to the recession is still present.

Tooth #23 was in a buccal prominent position, also presenting a cervical restoration in good maintenance. Adherent keratinized tissue apical to the recession was still present with small but still adequate thickness and high to perform a CAF (Figure 3). In accordance with Stefanini and coworkers [19], considering the interdental clinical attachment loss and the soft tissue loss, combined with the buccal malposition of the root, a CAF together with a CTG was planned.

**Figure 4:**
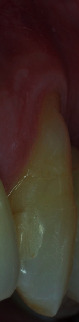
The composite restoration is reshaped at the maximum root coverage level.

After local anaesthesia, using articaine with adrenaline (1 : 100.000), the maximum root coverage (MRC) [20] was determined, and the present cervical restoration was shortened and smoothed at that level (Figure 4).

**Figure 5:**
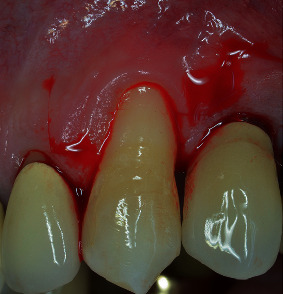
A trapezoidal flap is designed with vertical releasing incisions arriving just at the mucogingival junction.

**Figure 6:**
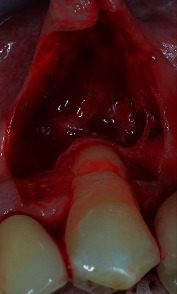
Two horizontal releasing incision are done: one deep, parallel to the bone crest and one, more extended, more superficial parallel to the oral mucosa (note the elasticity of the mucosa).

**Figure 7:**
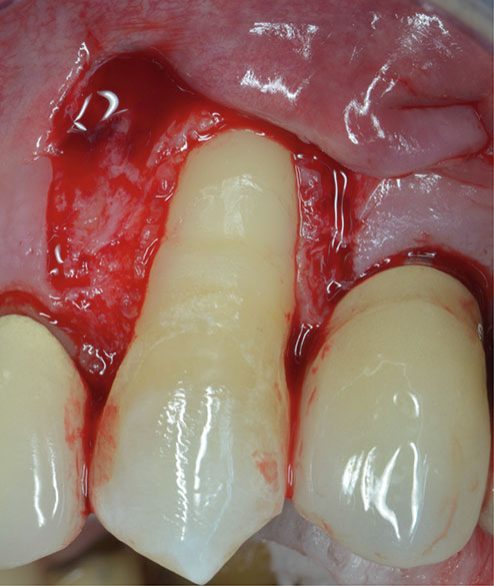
Root surface is treated with curettes and anatomical papillae deepithelialized.

**Figure 8:**
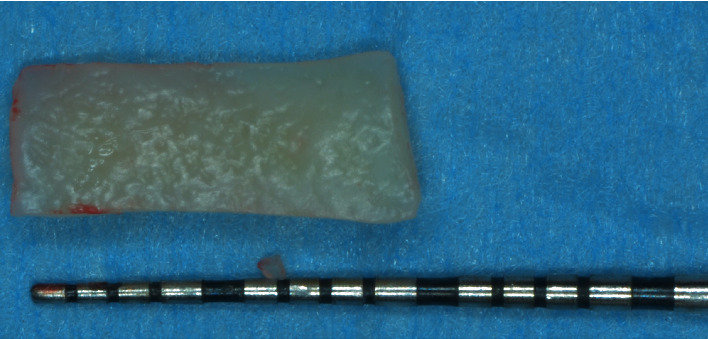
An epithelial-connective tissue graft is harvested. The height is 4 mm, and the length corresponds to the root width plus 3 mm.

**Figure 9:**
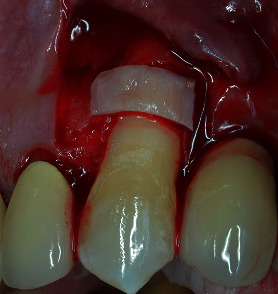
The connective tissue graft is attached with the fibrin-fibronectin glue to the root at the MRC level.

**Figure 10:**
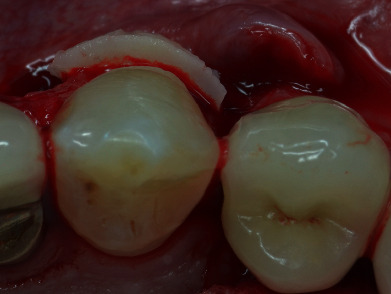
Note the thickness of the CTG bonded to the root.

**Figure 11:**
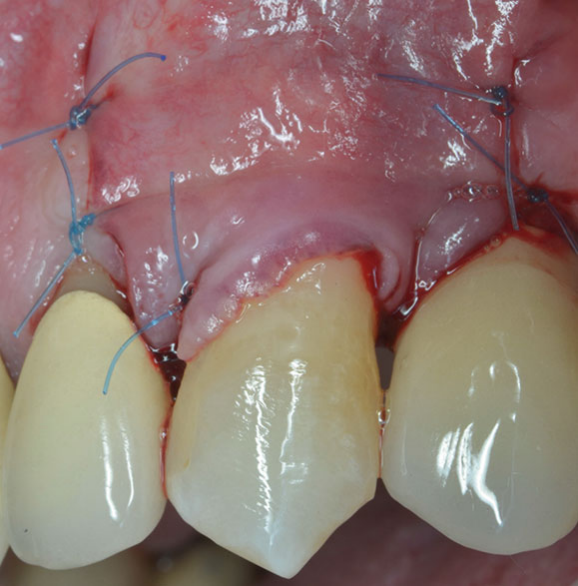
The flap has been coronally advanced and sutured.

The amount of gingival recession, plus 1 mm distance, was reported buccally and vertically from the top of the mesial and distal anatomical papillae, and at this level, a horizontal bleeding line of 3 mm was done with the top of the mini 15-c blade. From the angular point of such bevelled incision slightly divergent, two split thickness incisions were performed reaching the mucogingival junction (Figure 5). Afterwards, with the blade parallel to the oral mucosa, two split thickness surgical papillae were elevated, starting laterally and going out from the marginal sulcus. Split thickness surgical papillae ended when the coronal part of the free gingival margin was reached. With a proper elevator inserted into the sulcus, a full thickness central flap was elevated, exposing 3 mm of bone crest, apically to the anatomical root dehiscence. With a Gracey's minicurette root, the surface apical to the MRC was gently treated, removing the contaminated cementum. Then, a first deep horizontal releasing incision with the blade parallel to the bone crest was performed. After a second, more extended superficial horizontal incision was performed with the blade parallel to the oral mucosa, in order to mobilize the flap thanks to mucosa's elasticity, without cutting deeper structure, reducing in such way the possible bleeding and swelling (Figure 6). The anatomical papillae were deepithelialized (Figure 7). Anaesthesia was reinforced into the palate, and an epithelial-connective tissue graft was harvested. The dimension of the graft was 4 mm in height and in length, and the mesiodistal dimension was 1.5 mm exceeding in both side the avascular root surface in the area corresponding the MRC (Figure 8). The donor site was filled with a collagen sponge, and a compressive suture was performed reducing bleeding and stabilizing the sponge. Graft was gently de-epithelized with a blade. After washing the root surface with physiological solution, the fibrin fibronectin glue (Tisseel VH ®, Baxter, U.S.A.) was applied with two drops on the side of the graft facing the dental surface and the graft was immediately positioned on the root at the established level and maintained in such position with a slight finger pressure for 5 minutes (Figures 9 and 10). Then, after gently checking the stability of the graft, the flap was coronally advanced and sutured. Sutures started from the apical part of the vertical releasing incisions with circle point catching the periosteum when movable mucosa was laterally present. Finally, a sling suture, suspended around the cingulum was done, suturing the surgical papillae to the corresponding anatomical papillae (Figure 11).

The patient received antibiotic therapy, consisting of 1 g of amoxicillin plus clavulanic acid, starting from 1 day before surgery, twice a day, for 6 days; ibuprofen 600 mg was also prescribed to be taken twice a day for 2 days after surgery, then only if needed, and chlorhexidine spray to be used 3 times daily for 15 days.

**Figure 12:**
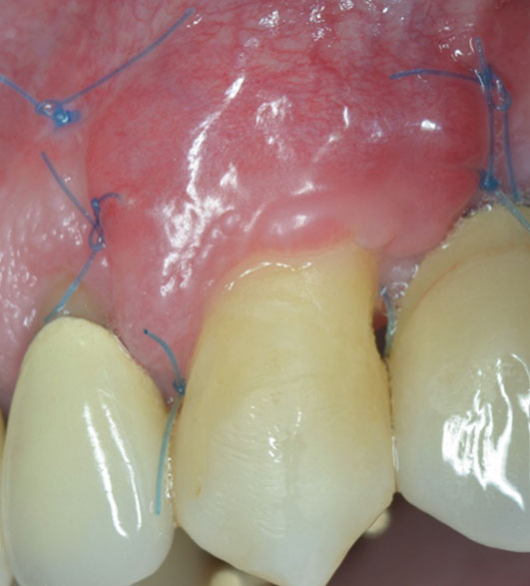
Healing of the flap after 15 days, at sutures removal.

**Figure 13:**
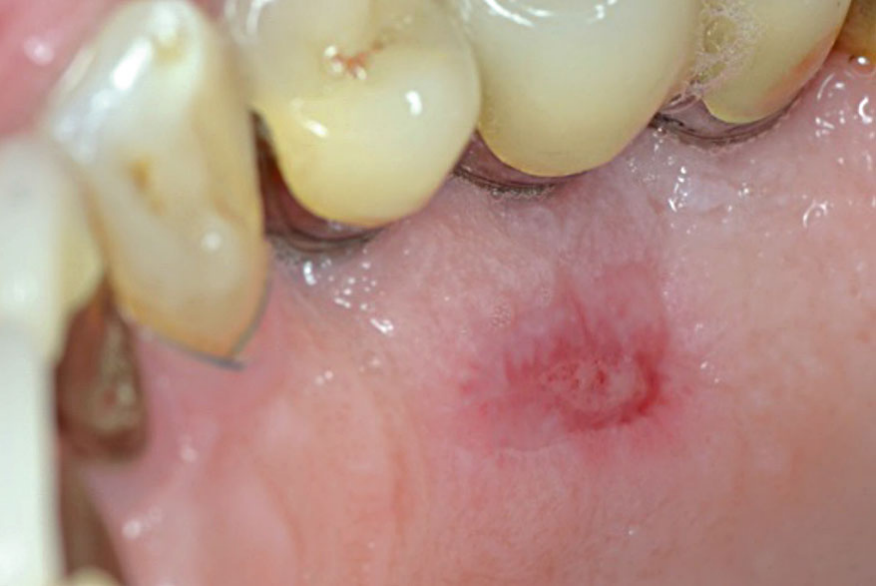
Palatal donor site healing after 15 days.

**Figure 14:**
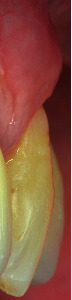
3-year lateral view (note the thickness of the soft tissue corresponding to the grafted area).

**Figure 15:**
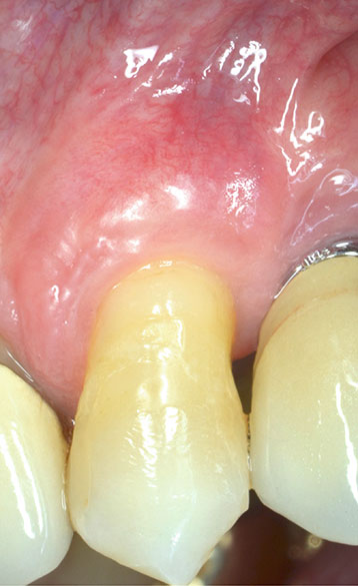
3-year buccal view (note the stability of the gingival margin, the partial root coverage achieved and the aesthetic blending of the area).

Healing period was uneventful. After 15 days, sutures were removed and both surgical areas appeared in good status (Figures 12 and 13). Patient was instructed not to brush for other 20 days, reducing chemically plaque accumulation; then, for the first month, it was possible to brush with an ultrasoft toothbrush, later using the classic soft toothbrush. He was enrolled in a supportive nonsurgical periodontal therapy every six months. At 3-year follow-up, free gingival margin was stable at MRC level, no buccal probing was recorded, and marginal soft tissue texture and blending were equivalent to the adjacent tissues. In addition, gingival thickness graft improved during these years, creating a good connective tissue protection to the underlying periodontal structures as well as a natural emergency profile of the anatomical crown (Figures 14 and 15).

## 3. Discussion

Coronally advanced flap with the addition of a subepithelial connective tissue graft has demonstrated to be effective in covering the root at the MRC, providing optimal clinical and aesthetic results [21, 22].

To the best of our knowledge, this surgical technique has never been reported. This modified, simplified bilaminar technique could enable clinicians to achieve several targets: to reduce the dimension of the palatal graft (the length particularly), to simplify the graft stabilization without using sutures, and to possibly promote an effective adhesion between the graft's connective tissue and the connective fibres in the dentin tubules. The proposed natural healing could determine the soft tissue thickness augmentation as well as the root coverage maintenance.

In this 3-year follow-up, we have noticed a proper tissue's augmentation, together with the reestablishment of the natural emergency profile of the affected tooth crown, similar to the goals usually obtained with the conventional technique, but in a simpler and possible more biological manner. Traditionally, the graft is sutured to the split thickness anatomical papillae or the adjacent recipient split area. Ideally, even if it is well positioned on the root, the graft is not very attached to the exposed root but just stretched on it. The fibrin-fibronectin human glue used have demonstrated to allow a real biological, chemical, and physical attachment with the collagen present in the dentin tubules, consequently improving the stability of the coagulum [23]. In addition, bonding the graft is much easier than suturing it. Thanks to these aspects, graft could have reduced dimension; it was 4 mm in height, and as reported in literature, there is no need to harvest graft covering the apical bone crest or all the radicular exposure in such a case [17]. In fact, in the original described technique, graft covering adjacent bone areas acted as an obstacle form blood supply between the flap and the recipient bed. This aspect could sometimes influence to early graft exposure and to obtain not appropriate aesthetic results [24]. The mesiodistal extension is also shortened. In case of sutures, 3 mm interproximal exceeding root exposure was recommended. In this case report, the dimension was reduced of the 50% (1.5 mm in each side), shortening the global length of 3 mm.

The abovementioned peculiarities improved the adaptation of the surgical papillae to anatomical, optimizing vascular exchange without interfering the healing and avoiding the post-op look of papillae too big implementing the aesthetic result in medium-term follow-up.

The adjunct of the fibrin-fibronectin glue to this bilaminar modified technique has demonstrated to be effective in reaching a proper graft stability, providing prompt clinical attachment and no mobility on the root, and reducing its length with fewer healing area of the palatal donor site. These advantages should be confirmed by a larger sample of patients, but they seem promising in terms of patient satisfaction and clinical results.

## Data Availability

The data supporting the results can be obtained by asking directly to dr. Michele Perelli.
